# Synthesis and Evaluation of Thiol-Conjugated Poloxamer and Its Pharmaceutical Applications

**DOI:** 10.3390/pharmaceutics13050693

**Published:** 2021-05-11

**Authors:** Muhammad Zaman, Sadaf Saeed, Rabia Imtiaz Bajwa, Muhammad Shafeeq Ur Rahman, Saeed Ur Rahman, Muhammad Jamshaid, Muhammad F. Rasool, Abdul Majeed, Imran Imran, Faleh Alqahtani, Sultan Alshehri, Abdullah F. AlAsmari, Nemat Ali, Mohammed Alasmari

**Affiliations:** 1 Faculty of Pharmacy, University of Central Punjab, Lahore 54000, Pakistan; shafeeq.rahman@ucp.edu.pk (M.S.U.R.); dr.jamshaid@ucp.edu.pk (M.J.); 2 Department of Pharmaceutics, Faculty of Pharmacy, The University of Lahore, Lahore 54000, Pakistan; sadafsaeed14@gmail.com (S.S.); rabiabajwa370@gmail.com (R.I.B.); 3 Oral Biology, Institute of Basic Medical Sciences, Khyber Medical University, Peshawar 59000, Pakistan; saeed.ibms@kmu.edu.pk; 4 Department of Pharmacy Practice, Faculty of Pharmacy, Bahauddin Zakariya University, Multan 60800, Pakistan; fawadrasool@bzu.edu.pk (M.F.R.); abdulmajeed@bzu.edu.pk (A.M.); 5 Department of Pharmacology, Faculty of Pharmacy, Bahauddin Zakariya University, Multan 60800, Pakistan; imran.ch@bzu.edu.pk; 6 Department of Pharmacology and Toxicology, College of Pharmacy, King Saud University, Riyadh 11451, Saudi Arabia; afalasmari@ksu.edu.sa (A.F.A.); nali1@ksu.edu.sa (N.A.); 442106674@student.ksu.edu.sa (M.S.A.); 7 Department of Pharmaceutics, College of Pharmacy, King Saud University, Riyadh 11451, Saudi Arabia; salshehri1@ksu.edu.sa

**Keywords:** poloxamer, thiourea, thiolation, mucoadhesion, drug release, in vivo analysis, in vitro dissolution studies

## Abstract

The current study was designed to convert the poloxamer (PLX) into thiolated poloxamer (TPLX), followed by its physicochemical, biocompatibilities studies, and applications as a pharmaceutical excipient in the development of tacrolimus (TCM)-containing compressed tablets. Thiolation was accomplished by using thiourea as a thiol donor and hydrochloric acid (HCl) as a catalyst in the reaction. Both PLX and TPLX were evaluated for surface morphology based on SEM, the crystalline or amorphous nature of the particles, thiol contents, micromeritics, FTIR, and biocompatibility studies in albino rats. Furthermore, the polymers were used in the development of compressed tablets. Later, they were also characterized for thickness, diameter, hardness, weight variation, swelling index, disintegration time, mucoadhesion, and in vitro drug release. The outcomes of the study showed that the thiolation process was accomplished successfully, which was confirmed by FTIR, where a characteristic peak was noticed at 2695.9968 cm^−1^ in the FTIR scan of TPLX. Furthermore, the considerable concentration of the thiol constituents (20.625 µg/g of the polymer), which was present on the polymeric backbone, also strengthened the claim of successful thiolation. A mucoadhesion test illustrated the comparatively better mucoadhesion strength of TPLX compared to PLX. The in vitro drug release study exhibited that the TPLX-based formulation showed a more rapid (*p* < 0.05) release of the drug in 1 h compared to the PLX-based formulation. The in vivo toxicity studies confirmed that both PLX and TPLX were safe when they were administered to the albino rats. Conclusively, the thiolation of PLX made not only the polymer more mucoadhesive but also capable of improving the dissolution profile of TCM.

## 1. Introduction

The development and evaluation of novel polymers have become the topic of interest for many years. Different polymeric excipients have been introduced to the pharmaceutical industry for the delivery of various active pharmaceutical ingredients (APIs). Such polymers, which can enhance the efficacy, pharmacodynamics, and pharmacokinetic characteristics of different APIs, are being widely investigated as novel drug carriers in pharmaceutical research. Successful biomedical applications of pharmaceutical materials encourage scientists and researchers to explore and discover more advanced ingredients with modifiable characteristics under certain conditions. The synthetic polymeric material can be premeditated in various structures to fulfill biomedical objectives. For that reason, the acquirement of comprehensive knowledge regarding biocompatible materials is of great importance. In this respect, the applied polymers, either from natural or synthetic sources, can be modified by considering their ultimate use, like the delivery of the drug and protein and peptide and chemotherapy. Among synthetic polymers, poloxamer has found a wide range of applications because of its triblock structure. Both water-soluble and lipid-soluble constituents are present in its structure, which grants exclusive properties, including thermos-sensitivity and micellar formation [1].

One of the most promising techniques is thiolation, which is used to impart enhanced mucoadhesive properties to the polymers. The mucoadhesive drug delivery system usually intermingles with the mucin components, which are present in the mucus of the mucosal membrane. The interaction of the mucoadhesive drug delivery system with the mucus membrane prolongs the residence time at the specific site and hence provides the drug with an opportunity to get entered into the systemic circulation, ultimately enhancing the drug’s bioavailability and therapeutic effect [2]. Thiolated polymers entitled “thiomers” are produced by the covalent interaction of thiol moieties to the backbone of polymers. The incorporation of such a thiol group is the main reason for the increased mucoadhesion of the polymers. Thiolated polymers are intrinsically employed as drugs [3,4]. The efficiency of thiomers largely depends on the nature and structure of the parent polymer. Usually, the mucoadhesive polymers are used for thiolation [5]. Different drugs are compounded with thiomers to increase their absorption across the mucus membrane, as there is an increase in the residence time of the drug in a specific part of the body due to prolonged adhesion. It has been found that upon thiolation, the mucoadhesive property of the polymer increases by 2–140 times [6]. Mucoadhesive drug delivery systems are important because they exhibit many advantages over other drug delivery systems, such as increased contact time with the site of absorption, improved drug permeability, and enhanced drug concentration in plasma circulation [7]. Another additional benefit of thiol-conjugated polymeric material is its ability to protect against the enzymatic degradation of certain drugs, like peptides and proteins [8]. Moreover, the thiomers have the potential to be utilized in the development of various drug delivery systems that are aimed at different routes of administration [9,10].

Tacrolimus (TCM) is an immunosuppressant that belongs to BCS class II and has a low solubility, a high permeability, and a poor bioavailability of 24% [11]. The purpose of the study was to achieve the successful thiolation of PLX, the evaluation of its physicochemical properties, the confirmation of its biocompatibility, and its application as a suitable pharmaceutical excipient in the development of a modified release compressed tablet of TCM.

## 2. Materials and Methods

### 2.1. Material

PLX and Ellman’s reagent (5,5dithio-bis-(2-nitrobenzoic acid) were purchased from Sigma-Aldrich GmbH Chemie, Germany. Thiourea, methanol, potassium chloride, acetic acid, hydrochloric acid (Merck, Darmstadt, Germany), and distilled water were obtained from the research laboratory of The University of Lahore. All the chemicals and reagents used were of analytical grade.

### 2.2. Thiolation of PLX

**Figure 1:**
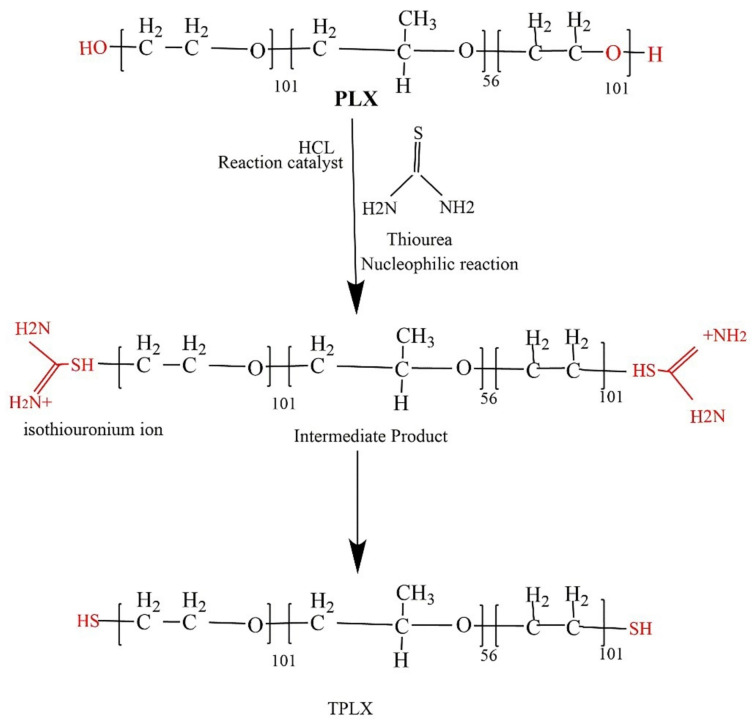
Schematic representation for the synthesis of TPLX using thiourea as the thiol donor.

The thiolation of PLX was done by using the method explained by Bernkop-Schnürch and co-worker in 2019 [3]. Briefly, the reaction was carried out in the presence of HCl as a catalyst. A 1% aqueous solution of PLX was prepared in a 100 mL glass beaker using distilled water under continuous stirring with hot plate magnetic stirrer at 500 rpm. Afterwards, 2 g of thiourea were mixed in the polymeric solution, followed by the addition of a catalytic amount of HCl (4–5 drops) to carry out the reaction. Initially, the thiourea, being a good nucleophile, and its nucleophilic reaction with HCl led to the production of an intermediate product. In the next step, the mixture was treated with alkali, which ultimately brought about the synthesis of TPLX (Figure 1). During the process, the reaction mixture was subjected to mixing for several minutes using a hot plate magnetic stirrer in order to get a homogenized mixture. Afterwards, it was kept in a water bath at 70 °C for 90 min. After the completion of the specified reaction time, the methanol was added into the mixture to cool it down. The resultant was filtered and washed again and again with HCl and distilled water to remove untreated thiourea. The final product was cooled at −80 °C and lyophilized at −47 °C and at 0.013 millibars of pressure to get a dried polymer. The freeze-dried polymer was kept in a closed container for further analysis. Both PLX and TPLX were used to prepare the modified-release tablets of TCM. A schematic representation of the synthesis of TPLX is given in Figure 1.

### 2.3. Physicochemical Properties of PLX and Thiolated PLX

#### 2.3.1. Solubility Studies and Swelling Index (SI)

PLX and TPLX were also checked for their aqueous solubility. Ten milligrams of TPLX and PLX were separately dissolved in 10 mL of water and checked for their aqueous solubility. To observe the residual insoluble material, the prepared solutions were filtered through No. 2 Whatman filter paper (Schleicher Schuell) with a pore size of 8 µm and a weight of 21 mg. After filtration, the filter paper was dried and weighed again to observe the weight gain due to the presence of any residue of the polymer on the paper’s surface. One gram each of thiolated and non-thiolated polymer was taken individually in the cylinders, and the initial volume was noted. Then, water was added to make the final volume 50 mL in both cylinders. It was covered with aluminum foil and left to stand overnight. After that, the final volume was recorded, and the swelling index of the polymers was calculated using the following formula:

Swelling index = (Final volume − Initial volume)/(Initial volume) × 100(1)

#### 2.3.2. pH of Aqueous Dispersion

The pH of thiolated and non-thiolated polymers was measured by preparing a 1% (*w*/*v*) aqueous dispersion. The probe of a pH meter (A120-Benchtop pH meter, BANTE instruments, China) was dipped in the aqueous dispersion until a constant pH was observed.

#### 2.3.3. Loss on Drying

One gram of both thiolated and non-TPLX was taken and allowed to dry separately in a digital moisture analyzer (MOC63U Unibloc Moisture Analyzer, Shimadzu, Quezon, Japan). The drying was continued until a constant weight was achieved and the LOD was recorded.

#### 2.3.4. Micromeritic Studies

Micromeritic parameters including bulk density, tapped density, Carr’s index, Hausner’s ratio, and the angle of repose were calculated for the analysis of the flow properties of the polymers [12]. The bulk density was calculated using the cylinder method. The weighed amount of polymer was poured in a graduated cylinder, and the volume was noted as the bulk volume to calculate the bulk density by comparing the mass and volume of the polymer. Similarly, the tapped density was determined by employing the tapping method, where a graduated cylinder with a known amount of polymer was tapped until a constant volume was achieved, which was noted as the tapped volume. This tapped volume was used to calculate the tapped density. The Carr’s index and Hausner’s ratio were calculated by using the values of bulk and tapped densities. Additionally, the angle of repose was determined by the fixed funnel method. 

#### 2.3.5. Determination of Thiol Content by Ellman’s Reagent Method

The standard linearity graph of the thiourea was plotted against the concentration on the x-axis and the corresponding absorbance on the y-axis. For this purpose, 2 mg of thiourea were accurately weighed and added in a test tube to prepare a 1 mL solution using a 0.5 M phosphate buffer of pH 8.0 as the solvent. Furthermore, the serial dilutions were prepared from this solution in the concentration range of 10–100 μg/mL. The absorbance of the prepared dilutions was determined by using a UV–visible spectrophotometer (PG Instruments T80, Leicestershire, United Kingdom) at 280 nm.

The thiol contents of TPLX were determined by using Ellman’s reagent technique [13]. In the 1st step, a 2% *w*/*v* solution of the polymer conjugate was prepared by dissolving it in a sufficient volume of deionized water. After that, 1350 μL of 0.1 M PBS at pH 7.4 were prepared. Then 150 μL of Ellman’s reagent were prepared by dissolving 3.96 mg of reagent in 10 μL of PBS, and 150 μL of polymer conjugate were added to 1350 μL of deionized water. The mixture was incubated at room temperature for 90 min, and then its absorbance was measured at a wavelength of 412 nm with a UV spectrophotometer [14]. The amount of the thiol group attached to the polymer was determined by a thiourea calibration curve.

#### 2.3.6. Fourier Transformed Infrared Spectroscopy (FT-IR)

FT-IR studies were performed to confirm the thiol modification of PLX. Both PLX and TPLX were taken, and the samples were scanned by using FTIR (Agilent Carry 360 FTIR, United States) in the range of 500–4000 cm^−1^ [15].

#### 2.3.7. Surface Morphology Studies

The surface appearances of the prepared solid dispersion of thiolated and non-thiolated polymers were done by SEM. A small amount of powder was placed on the stage of SEM (EVO LS 10 Zeiss, Germany), and photographs were taken using a lens at a 1000X magnification power [16].

#### 2.3.8. X-Ray Diffractometry (XRD)

An X-ray diffractometer (JDX-3532 JEOL Japan) was used to observe the impact of thiolation on the crystalline nature of PLX. An XRD diffractogram was plotted between the angle of diffraction (2θ) and the counts, and scanning was performed between 5 and 50º under a tube voltage of 45 kV and a tube current 40 mA [17].

#### 2.3.9. Acute Toxicity Studies of PLX and TPLX

Acute toxicity studies of PLX and TPLX were carried out according to OECD guidelines by administering a single dose of the individual polymers to the animals as 50, 300, and 2000 mg/kg body weight of each animal [18]. Fifteen albino rats of an average weight of 150–200 g were selected from the animal house of the University of Lahore and kept in clean cages with food and water supply. The animal study was approved by the Institutional Research Ethics Committee of The University of Lahore under project file no. IREC-2019-101, dated: September, 5, 2019. 

#### 2.3.10. Preparation of Test Animal

Fifteen (15) rats were kept in clean cages in the laboratory for one week to acclimatized to the environment. These rats were then divided into 3 groups. Each group had 5 rats. Groups I and II were titled for the administration of doses of PLX and TPLX, while Group III was used as a control group and was only administered food and water. Afterwards, the animals were subjected to acute oral and dermal toxicity studies. 

#### 2.3.11. Preparation of Dose

According to OECD guidelines, the dose was prepared according to the weight of each rat and moistened with water before administration. According to the guidelines, the volume of dose should not be greater than 1 mL/100 g. The single dose given for acute toxicity was up to 2000 mg/kg of the animal. Rats were observed for the first 4 h and then after the 3rd, 7th, and 14th days of the study, and the number of rats that survived after 14 days of study was noted. On the 15th day, the animals were sacrificed for further analysis.

#### 2.3.12. Physical Examination

Rats were examined once daily for health and any response to treatment, like changes in skin, hair, eyes, mucus membranes, and behavior, as well as convulsion, saliva production, diarrhea, sleep disorder, and coma, for 14 days.

#### 2.3.13. Skin Irritation and Dermal Toxicities

The animals were tested for skin irritation; for this purpose, hair was removed with a razor from the skin, and 500 mg of thiolated and non-thiolated polymers were individually applied to the naked skin. Then, rats were observed on a daily basis for skin irritation and any other sort of dermal incompatibility [19]. Animals were arranged into 3 groups. One group served as a control group, while the other 2 groups were treated with thiolated and non-thiolated polymers. Three percent solutions of PLX and TPLX were individually applied to the skin of the respective animals. After the application of a 3% solution, the skin was taped with gauze and observed for different time intervals. After 24 h, the gauze was removed and hair growth was observed until the 15th day.

#### 2.3.14. Bodyweight, Food, and Water Consumption

The body weight of rats of all the groups was recorded before and after the administration of the dose. After dosing, the weight was measured on the 7th and 14th days of the study.

#### 2.3.15. Hematological and Biochemical Examination

Hematological and biochemical examinations were done on all the animals used in the study. Blood was taken from the posterior vena cava of the rat under anesthesia, and the samples were examined for hematological observations, including Hb, WBCs, RBCs, and platelet count. Serum was analyzed via liver function tests (LFTs), renal function tests (RFTs), and lipid profiling.

#### 2.3.16. Relative Organ Weight (ROW)

On the 15th day, all albino rats were sacrificed. Three vital organs—the kidney, liver, and heart—were examined for the presence of any abnormality and lesion. These organs were then removed and weighed, and the relative organ weight was calculated by using the following formula [20].

ROW = (Absolute organ weight (g))/(Body weight of mice on sacrifice day (g)) × 100(2)

Then, organs were preserved in a 10% formalin solution for histopathological investigation.

### 2.4. Preparation of Modified-Released Tablet of TCM

The direct compression method was used to prepare the tablets of TCM while using PLX and TPLX as polymers. The other excipients that were used in the study were talc (lubricant), magnesium stearate (glidant), lactose (diluent), polyvinyl pyrrolidone (binder), avicel at pH 102 (flow modulator), and aspartame (sweetener). All the ingredients were accurately weighed and passed through a No. 40 sieve. After that, all the excipients were added one by one into the mortar and pestle and mixed for 30 min to confirm uniform mixing. Finally, the powder blend was directly compressed by using a single punch compression machine (Table 1).

### 2.5. Calibration Curve of TCM

A stock solution of TCM was prepared by dissolving 2 mg of TCM in 1 mL of a 7.4 pH PBS solution in a volumetric flask with a capacity of 50 mL. The standard solution was prepared by diluting 0.5 mL of stock solution in 9.5 of the solution. Then, serial dilutions were prepared in the concentration range of 10–100 µg/mL. The absorbance of all the dilutions was taken at 253 nm using a UV spectrophotometer, and a calibration curve of TCM was drawn.

### 2.6. Post-Compression Studies of PLX- and TPLX-Based Tablets of TCM

The following post-compression tests were performed on TCM modified-release tablets.

#### 2.6.1. Thickness and Diameter

The diameter and thickness of tablets of F1 and F2 were measured using a vernier caliper. Twenty tablets of each formulation were selected, and then the average thickness and diameter of these 20 tablets were calculated [21].

#### 2.6.2. Tablet Hardness and Friability Test

The ability of tablets to resist any breakage during storage, transportation, and handling is called hardness. A Monsanto hardness tester was used to measure the hardness of all formulations, and it was calculated as kilogram per centimeter square [22]. On the other hand, for the determination of friability, 10 tablets of each formulation after weighing and dusting were placed in a Friabilator at 100 rpm for 4 min (25 rpm/min). Tablets were taken out, and the total weight of the tablets was recorded. Friability was calculated by using the following equation [23].

Friability = (Initial weight − Final weight)/(Initial weight) × 100(3)

#### 2.6.3. Weight Variation Test

The weight variation test was performed by individually weighing 20 tablets of each formulation of F1 and F2 on an analytical balance and then taking the average weight of these 20 tablets. The final values were compared to the USP pharmacopeia limits [24].

W.V = (IW − AW)/AW × 100(4)

#### 2.6.4. Disintegration Test

A disintegration test was carried out in the disintegration apparatus (Microprocessor Disintegration Test Apparatus, JL-DTA-2213). Six tablets from each formulation (F1 and F2) were placed in the basket, which was filled with distilled water and maintained at temperature 37 ± 2 ºC. In the end, the basket was lifted and checked for disintegration. The disintegration time of 6 individual tablets was determined, and the average time was recorded [25].

#### 2.6.5. Swelling Index

The swelling index was calculated to determine the ability of the tablet to absorb and retain the liquid. The weight of each tablet was measured (W1) and then separately placed on slides. The weight of the slide was tarred and placed in a petri dish. Five milliliters of PBS at 7.4 pH was added, and swelling studies were performed. The glass slides containing tablets were removed from the petri dish, the excess of the solution was removed with the help of filter paper, and then the weight was measured after different time intervals (15 min, 30 min, 45 min, 1 h, 2 h, 4 h, 6 h, 8 h, and 24 h) until a constant weight was achieved. The swelling index was measured by using the following equation.

SI = (W2 − W1)/W1 × 100(5)

where W1 is the initial weight and W2 is the final weight [26].

#### 2.6.6. In Vitro Dissolution

The paddle method was used to determine the release rate of the drug from thiolated and non-thiolated polymer-based tablets of TCM. A 900 mL 7.4 pH buffer solution was used as the dissolution media. Tablets were transferred and continuously stirred at 50 rpm under the maintained temperature condition of 37 ± 5 °C. Next, 5 mL samples were withdrawn after specified time intervals of 2 min, 5 min, 10 min, 15 min, 20 min, 30 min, 1 h, 2 h, 4 h, 6 h, and 8 h. The solution was replaced with fresh dissolution media to maintain the sink condition. The absorption of these samples was recorded at 253 nm using a UV–visible spectrophotometer. A curve of the percentage of drug release against time was drawn to observe the drug release pattern [27].

#### 2.6.7. Drug Content

A standard solution of TCM was prepared by dissolving 4 mg of the pure drug in a phosphate-buffered solution (PBS) of pH 7.4 in 100 mL volumetric flasks. On the other hand, ten (10) tablets of each formulation of TCM were taken and crushed individually, and the mass of the crushed powder equivalent to 4 mg of the drug was taken and dissolved in a 100 mL volumetric flask containing PBS of pH 7.4. These solutions were analyzed on a UV spectrophotometer at a wavelength of 253 nm, and then the dissolution factor was applied and drug contents were calculated by the following formula.

% Drug content = (Absorbance of sample)/(Absorbance of standard) × (Concentration of standard)/(Concentration of sample)(6)

#### 2.6.8. Mucoadhesion Strength

**Figure 2:**
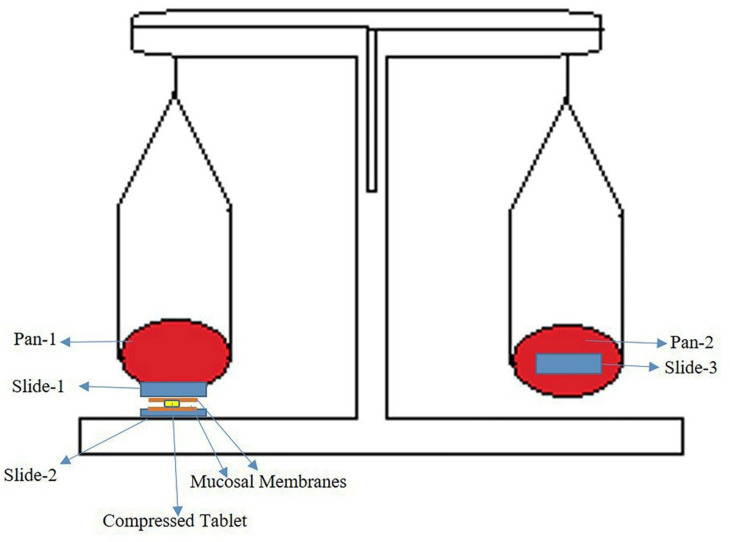
Graphical representation for the determination of mucoadhesion strength of tablets by sandwiching them between glass slides mounted with pieces of mucosal membranes.

Prepared tablets were subjected to the determination of mucoadhesion strength by using a modified physical balance that consisted of two pans. Three glass slides were taken and attached to the pan in such a way that one slide was stuck to the bottom of the pan and the second one was right beneath that pan. The third slide was placed on the other pan to tare the balance (Figure 2). Mucosa pieces of rabbit were taken and cut into pieces. Mucosa pieces were then placed on the glass slides. A compressed tablet was taken and individually placed between the pieces of the mucosa that was attached to slides 1 and 2. The tablet was gently pressed between the pieces of the mucosa to properly attach. A force of 50 gm was applied to the second pan, and this was gradually increased until slides 1 and 2 were separated from each other. The mucoadhesion strength was calculated for each formulation and reported [28].

### 2.7. Statistical Analysis

Statistical analysis was conducted to evaluate the drug release profiles of F1, F2, and marketed products. For this purpose, an ANOVA was applied followed by Tukey’s multiple comparison test at a level of significance of 95%.

## 3. Results

### 3.1. Physicochemical Evaluation of PLX and TPLX

The physicochemical properties of PLX and TPLX were evaluated, and a comparison was made between thiolated and non-thiolated polymers.

The solubility of thiolated and non-TPLX was checked in water. Both PLX and TPLX are soluble in water. The weight of the filter paper after drying was approximately the same (21.003 mg) as observed before filtration (21 mg).

The swelling index of both PLX and TPLX was checked, and it was observed that they did not show considerable swelling abilities. When the polymers were exposed to water for the swelling study, they initially showed swelling (≈3.03%) to some extent but later dissolved, making it difficult to further measure the swelling index.

Similarly, for the determination of pH, 1% *w*/*v* solutions of PLX and TPLX were separately prepared. The outcomes of the study showed that the pH values of PLX and TPLX were 7.85 and 6.68, respectively. On the other hand, the loss on drying for PLX and TPLX were calculated to be 1.4% and 11.2%, respectively (Table 2).

### 3.2. Micromeritics

The micromeritic properties of PLX and TPLX were observed and compared to check the flowability of the polymers. The bulk density and tapped density of PLX were calculated to be 0.466 and 0.523 cm^3^/mL, respectively. On the other hand, the Hausner’s ratio and Carr’s index were found to be 1.16 and 10.9, respectively. The value of the angle of repose for PLX was found to be 25.78. These results confirmed that the flow properties of PLX were good, whereas the bulk density and tapped density of TPLX were found to be 0.505 and 0.566 cm^3^/mL. The Hausner’s ratio, Carr’s index, and angle of repose were calculated to be 1.15, 10.7, and 21.80, respectively, exhibiting excellent flow properties (Table 3).

### 3.3. Thiol Contents

The thiolation of the polymers was done by reacting PLX with thiourea. A thiol group attached to the polymeric backbone was confirmed by Ellman’s reagent method, as analyzed on a spectrophotometer. Ellman’s reagent was used to determine the thiol content present in the polymers. The thiol content of TPLX was found to be 20.625 µg/g of the polymer. The presence of the thiol group confirmed the successful thiolation.

### 3.4. Calibration Curve of Thiourea

Different dilutions of thiourea, ranging from 10 to 100 µg using a 2 mg/mL stock solution, were prepared. These dilutions were analyzed on a UV–visible spectrophotometer at a wavelength of 280 nm. A linear calibration curve was obtained by plotting concentration µg/mL along the x-axis and absorbance along the y-axis (Supplementary Figure S1).

### 3.5. FTIR Studies

**Figure 3:**
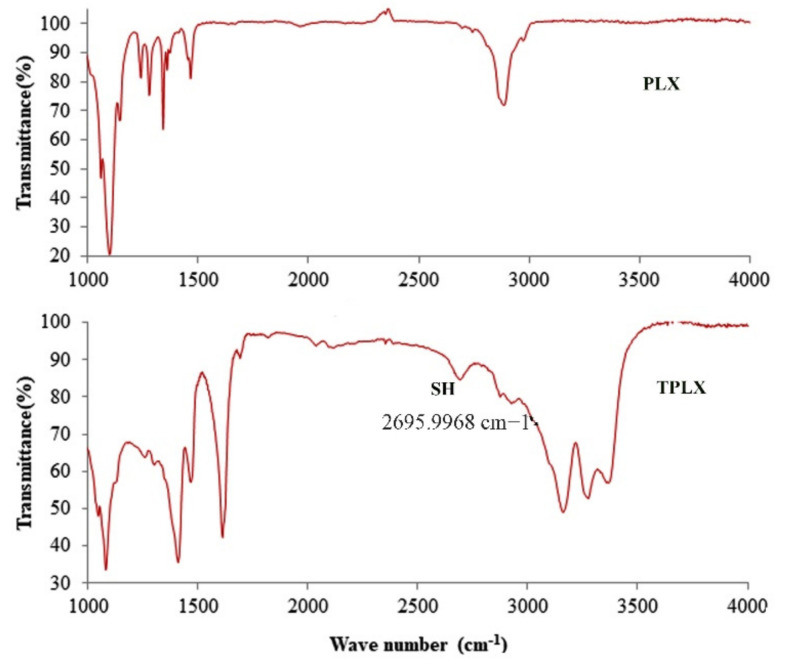
Comparative illustration through FTIR scans of both LX and TPLX, confirming the occurrence of chemical changes in the structure of PLX. The appearance of the characteristic peak in the scan of TPLX at 2695.9968 cm^−1^ indicated the successful modification of the polymer.

Thiolated and non-thiolated polymers were subjected to FTIR analysis, which revealed the successful thiolation of polymers. In comparison to PLX, TPLX showed a characteristic peak at 2695.9968 cm^−1^ that confirmed the attachment of a thiol moiety to the polymer. A C–O stretch was seen at 1079.9415 cm^−1^, and an N–H bend was observed at a wavenumber of 1610.2699 cm^−1^, which was due to the amine group of thiourea (Figure 3).

### 3.6. Scanning Electron Microscope (SEM)

**Figure 4:**
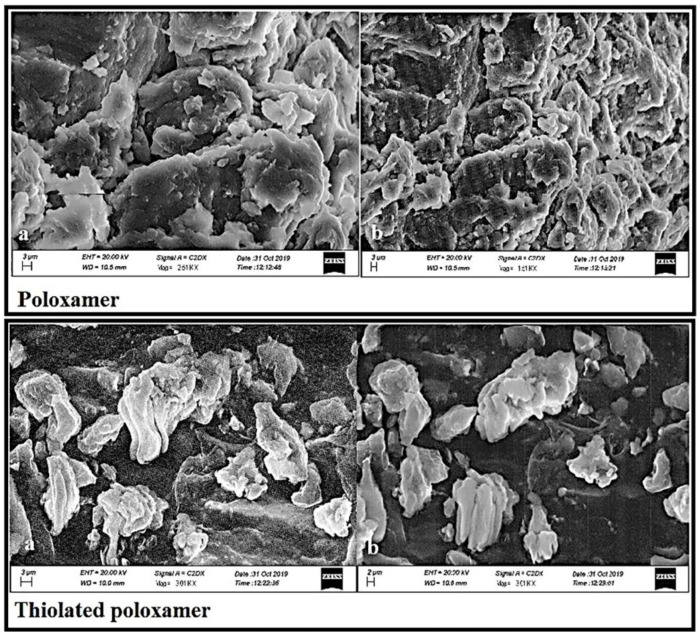
SEM images describing the surface morphology of PLX and TPLX at different magnifications (**a**) 1000X and (**b**) 500X. A change in the apparent surface morphology of TPLX can be observed, thus indicating the chemical modification of PLX.

SEM images of PLX illustrated a crystalline and shiny surface, whereas when the thiol group was attached to the polymer, distinct changes in the surface were noticed. Apparently, they seem to be more compact but irregular in shape. Figure 4 confirms the observed changes in the surface morphology of PLX.

### 3.7. XRD Studies

**Figure 5:**
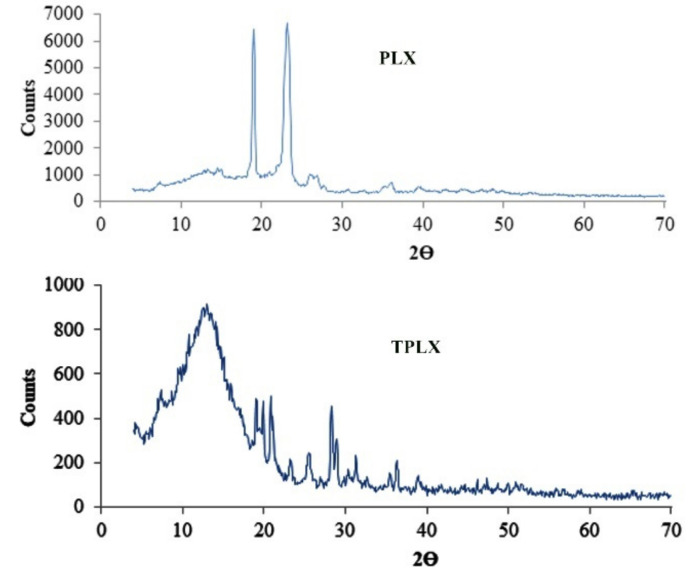
XRD diffractogram of PLX and TPLX exhibiting various peaks between 20 and 40. Sharp peaks present in the structure of PLX was found to be diminished in the graph of TPLX, indicating a noticeable change in its structure upon thiolation.

The effect of thiolation on the crystalline structure was observed using an XRD diffractogram. The XRD diffraction spectra of PLX exhibited sharp peaks at diffraction angles (2θ) 180 and 220 and a less intense peak at 260. However, peaks were also observed in the diffractogram of TPLX, but their intensity was reduced to a greater extent (Figure 5).

### 3.8. Acute Toxicity Studies of PLX and Thiolated PLX

#### 3.8.1. Physical Examination

After treatment, the physical examination showed that the animals were found to be normal. The skin was intact, and normal hair growth and eye color were noted. After the oral administration of the contents, no abnormal behavior was observed during the study because normal gate, food intake, and water drinking were witnessed. Moreover, neither diarrheal conditions (as normal stools have been observed) nor any other abdominal discomfort was observed.

#### 3.8.2. Skin Irritation and Dermal Toxicity

The albino rats did not show any toxic effects—not even skin irritation or signs of dermal toxicities were observed. The outcomes revealed that both the treated and normal groups exhibited similar findings, as no skin irritation or any sort of dermal toxicities from the 1st hour to the 14th day of the study were found.

#### 3.8.3. Body Weight, Food, and Water Consumption

All the groups except for the control group were orally administered with a 2 mL solution of modified and unmodified polymers to perform a single-dose toxicity study.

Bodyweight of control and tested rats were observed before dosing, and then after 1, 3, 7, and 14 days right before their necropsy procedure. A gradual weight change was observed for tested animals in the first seven days. Later on, the weight continued to improve until the last day (Table 4).

#### 3.8.4. Hematological and Biochemical Examination

All the data obtained from blood chemistry and hematology indicated no abnormal values, hence proving that no toxic effect was observed during the acute toxicity study arranged for 14 days. Blood profiles observed during the study included complete blood count, renal profile, and kidney profile. The results disclosed that both PLX and TPLX were safe for in vivo use.

#### 3.8.5. Necropsy

Necropsy was done on the 15th day on all rats. To check any kind of abnormal effects, three organs, i.e., kidney, heart, and liver, were observed. The necropsy showed that all the organs were normal in appearance.

#### 3.8.6. Relative Organ Weight

After dissection, the relative organ weight of animals was calculated. All the weights noted were in the normal range and expressed as the mean of *n* = 3 (Table 5).

#### 3.8.7. Histopathological Evaluation

**Figure 6:**
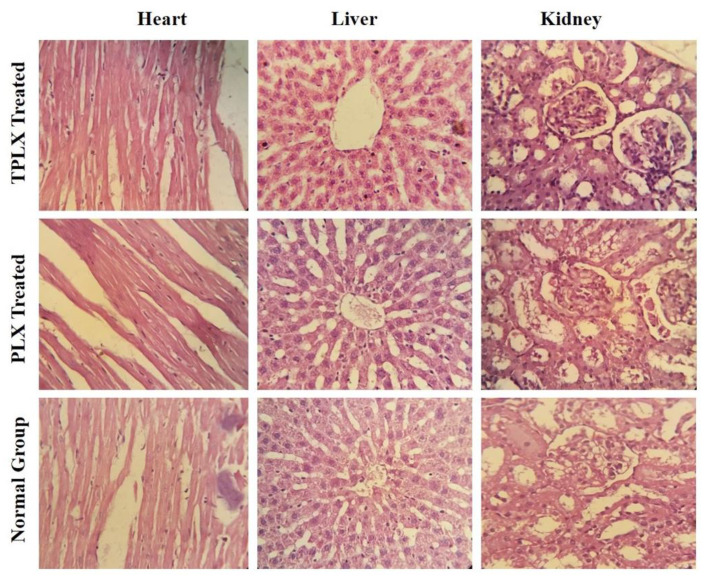
Histopathological evaluation of control group, PLX-treated group, and TPLX-treated group, illustrating the safety profile of modified and unmodified polymers. All the vital organs had normal physiological and anatomical features.

The three vital organs were selected as the heart, liver, and kidney from all groups. After washing with normal saline preserved in a 10% formalin solution for further histopathological investigation, vital organ tissues were stained with hematoxylin and eosin for evaluation purposes (Figure 6). Images of vital organs of control and treated animals were taken at a magnification power of 400X. Histopathological evaluation showed that the observed vital organs (heart, liver, and kidney) of all the animals, placed in different groups, were found to be unaffected. The cardio-myocytes, hepatic cells, and renal tissues of both treated groups were comparable to those of the control group. No signs of toxicity, swelling, inflammation, or damaged cells were noticed during histopathological observation. Hence, the findings confirmed that both modified and unmodified polymers might be employed in the development of oral dosage forms for the delivery of TCM.

### 3.9. Preparation of Modified-Release Tablets of TCM

Modified release tablets of TCM using PLX were prepared by the direct compression method. A net weight of 150 mg, amounting to 4 mg of the drug, were kept for the formulation. The other included excipients were polyvinyl pyrrolidone as a binder, magnesium stearate as a glidant, aspartame as a sweetener, avicel at a pH of 102 as a diluent, and talc as a lubricant. 

### 3.10. Calibration Curve of TCM

Different dilutions of TCM, ranging in concentration from 10 to 100 µg/mL using a 2 mg/mL stock solution. These dilutions were analyzed on a UV–visible spectrophotometer at a wavelength of 253 nm. A linear calibration curve was obtained by plotting concentration (µg/mL) along the x-axis and absorbance along the y-axis. The coefficient of correlation (R^2^) was 0.9922, describing the good linearity of the standard graph (Supplementary Figure S2).

### 3.11. Post-Compression Tests

TCM tablets of the F1 and F2 formulations were tested for thickness, diameter, hardness, friability, weight variation, disintegration, in vitro drug release, swelling index, and drug content.

#### 3.11.1. Diameter and Thickness

Thickness and diameter were measured using a Vernier caliper. The mean diameters for the F1 and F2 formulations were found to be 1.1 ± 0.08 and 1.1 ± 0.073 mm, respectively. Thickness was found to be 0.5 ± 0.009 mm for F1 and 0.6 ± 0.147 mm for F2. The negligible value of standard deviation showed that the tablets of both the formulations were uniform in thickness and diameter.

#### 3.11.2. Hardness and Friability Test

A Monsanto hardness tester was used to determine the mechanical strength of the PLX- and TPLX-based tablets. The hardness was found to be 4.1–4.25 ± 0.234 kg/cm^3^ for F1 and 4.1–4.2 ± 0.132 for F2. Similarly, the tablets were found to be of good mechanical strength, as the friability was found to be less than 1% for both formulations.

#### 3.11.3. Weight Variation Test

Twenty (20) tablets from each formulation (F1 and F2) were used to report variation among the weight of the tablets. The average weights of the PLX and TPLX-based tablets were found to be 148–160 and 145–162 mg, respectively. The average weight was calculated and compared to the individual weight of the tablets. The results satisfied the USP specification of ±10%.

#### 3.11.4. Disintegration

Six (6) tablets of formulations F1 and F2 were allowed to disintegrate in the disintegration apparatus. The time taken by the tablets to completely disintegrate was noted and compared. The F1 and F2 formulations took 9 ± 0.07 and 10 ± 0.08 min, respectively, to disintegrate.

#### 3.11.5. Swelling Studies

**Figure 7:**
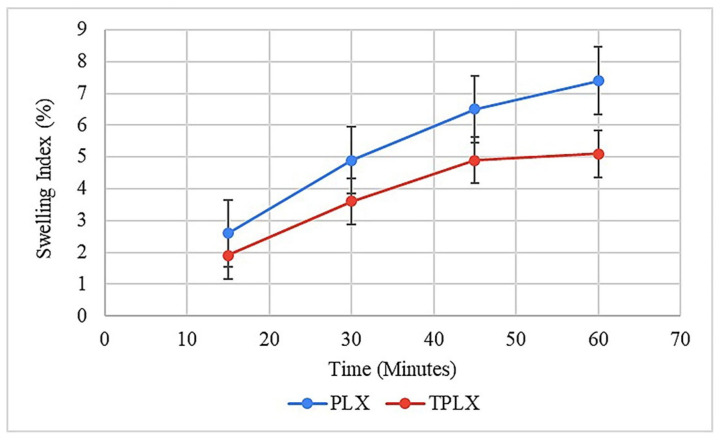
Swelling index of PLX- and TPLX-based tablets of TCM.

The potential of the polymer to absorb and retain moisture was found via swelling studies. Tablets based on PLX and TPLX showed gradual increases in swelling. Swelling was observed until the constant weight was achieved. It was observed that after an hour, the tablets were dispersed. The maximum swelling values of the PLX- and TPLX-based tablets were found to be 5.9% and 7.6%, respectively. Figure 7 illustrates the significant difference between the swelling behavior between the two different formulations, where F2 was swelling more rapidly. It might be the reason that TPLX has a greater ability to retain moisture contents and a faster rate of hydration, thus allowing for comparatively greater swelling [29].

#### 3.11.6. In Vitro Drug Release

**Figure 8:**
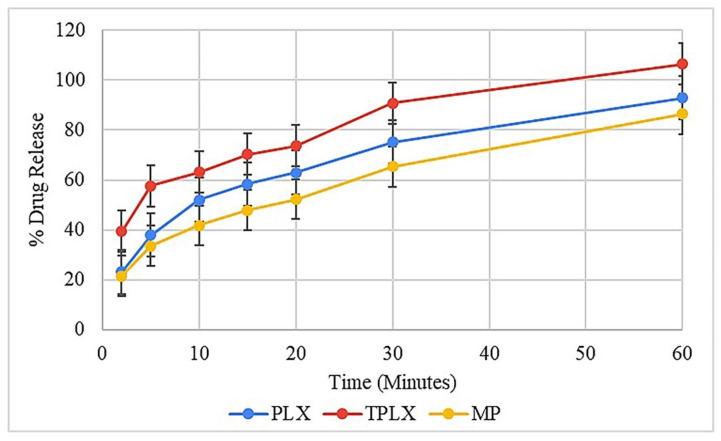
Describing the significant difference (*p* < 0.05) in release of the drug from PLX- and TPLX-based formulations.

PLX is considered to be an effective solubilizer and dissolution profile enhancer, and the literature has shown evidence of its properties [30,31]. Here, it was observed that the PLX-based formulation (F1) released more than 60% of the drug in the initial 15 min of the study. On the other hand, TPLX exhibited a more rapid response and a significantly improved dissolution output. As illustrated in Figure 8, F2 released more than 70% of incorporated drugs in a similar period. Furthermore, the prepared formulation was compared with that of the marketed product, and similar types of outcomes were observed. The findings of the study advocated that, by incorporating the thiol group, the dissolution-enhancing abilities of PLX could be further improved to a significant extent. ANOVA statistically proved (*p* < 0.05) that TPLX had a better dissolution enhancement impact than PLX.

#### 3.11.7. Drug Content (DC%)

Formulations F1 and F2 were checked for their % drug content. The results showed that the % drug content for F1 and F2 were 95.89% ± 1.2 and 97.46% ± 1.85%, respectively, which were under the USP specification range (85–115%) (Table 6).

#### 3.11.8. Kinetic Modeling

Different kinetic models like zero order, first order, Higuchi, and Korsmeyer Peppas were applied to find the release kinetics of formulations F1 and F2. In the F1 and F2 formulations, the release kinetics were not dependent on the initial concentration of the drug, as the R^2^ of the zero-order model (0.9157) was greater than that of the first order model (0.760) for F2 and F1. Hence, it can be considered that TPLX could also help to control the release of the drug. The best fit model was found to be the Korsmeyer Peppas model, as it got the highest R^2^ value of the other applied models. Higher values of R^2^ for the Higuchi model (F1: 0.8037; F2: 0.6878) confirmed the diffusion-type release pattern of the drug from the prepared tablets. The correspondent values of ‘n’ suggested that the release of the drug did not follow Fick’s law of diffusion (Table 7).

##### Mucoadhesion Strength of Formulations (F1 and F2)

The results of the study indicated that the mucoadhesive strength of TPLX-based tablets was greater than that of PLX-based tablets. The incorporation of an –SH moiety in PLX led to an increase in mucoadhesion strength from 0.99 to 2.95 N (Table 8).

##### Statistical Analysis

Statistical analysis showed that the effect of TPLX on drug release was significantly different from that of PLX and the marketed product on the release of TCM from compressed tablets (Table 9).

## 4. Discussion

Thiolation is considered to be an approach that can modify the characteristics of the polymer. Here, thiol modification was confirmed by the presence of the considerable amount of the thiol contents (20.625 µg/g of the polymer) in the modified polymer. The claim of successful thiolation was further strengthened by the emergence of a characteristic peak at 2695.9968 cm^−1^ in an FTIR scan of TPLX. Different characteristic changes were observed after the incorporation of thiol moieties in the structure of PLX, as detected in SEM, where intensive peaks confirming the crystalline structure were found to be converted into comparatively broader peaks. The decrease in the intensity of the crystallinity were predicted by the x-ray diffractogram. Poor swelling properties were displayed by PLX and its modified form, which might have been due to the presence of linear chain monomeric units in the structure of PLX.

These findings were comparable to those of various reported studies, where researchers have disclosed that thiolation might not significantly improve the swelling index of certain polymers. Bernkop-Schnürch and his co-worker observed the swelling index of tablets prepared with thiolated and non-thiolated carboxymethyl cellulose, and they revealed that there were no considerable differences in the characteristics of the tablets [32]. Furthermore, in another study that was based upon the modified and unmodified chitosan, the author statistically evaluated the swelling index by using a Student’s t-test. He observed that there was no noticeable difference in the swelling profile of both of the polymers [33]. The conversion of PLX into TPLX markedly changed the behavior of the polymer, as well as its impact on the release of the drug. The formulation based on TPLX showed a more rapid drug release than PLX, as confirmed by the statistical analysis (*p* < 0.05), where a significant difference was observed in the amount of the drug released (Table 9). Similarly, the tablets were found to be rapidly disintegrating, and this might have been the reason that the maximum amount of the drug was released in 1 h. This short period might not have been sufficient for the tablets to get attached to the mucus membrane, allowing the mucoadhesive properties of the thiolated polymer to get in and play their role. However, by adjusting the suitable concentrations of the polymer, a better sustained effect could be produced, which may have allowed the tablets to get attached to the mucosal surface. This interaction of the dosage form may have provided the drug an opportunity to get permeated across the mucus membrane in a greater concentration from a specific site, hence benefiting an increased permeation and leading to an enhanced bioavailability and therapeutic effects. 

The mechanism of drug release was predicted to be independent of the initial concentration of the drug in tablets, which was the indication that PLX might be used for the development of a controlled release drug delivery system. Another important point that was noticed was the diffusion-type drug release pattern. Though the pattern was non-Fickian, it could be helpful to control the release of the drug by controlling the thickness of the diffusive layer, which is dependent on the concentration of the polymer. In the same way, the tablet assay showed that each dosage form contained a sufficient amount of the drug to satisfy the official limits of the drug contents. It proved that PLX and TPLX have satisfactory drug loading capacities. The property of good drug loading capacity was might be due to the fact that PLX is an amphiphilic polymer [34], and it can therefore also hold poorly water-soluble drugs, such as TCM. After toxicity study evaluation, the findings established the fact that the addition of a thiol group might not cause any sort of toxic effects in the body. It was an important outcome that could help to suggest the initiation of in vivo studies of the prepared formulation. Similarly, another study also found that the thiomer could be safe for in vivo use [35]. PLX has also been widely studied by other researchers to confirm its biocompatibilities, and several studies have confirmed that it is one of those excipients that can be safely used as a carrier for drug administration [36,37].

The oral and dermal compatibilities of the polymers have opened a gateway for their diverse applications. Such modified polymers can be effectively used for both the oral and topical administration of the drug, as thiolation can enhance adhesion strength from 2 to 140 folds, providing a better contact time for the formulation on the skin [38].

## 5. Conclusions

The objectives of the study were productively achieved, as PLX was invariably converted into TPLX and proved to be a key potential carrier of TCM. These results showed the polymers’ reliable compatibility and sufficient loading capacity of the drug. The polymer was found to be non-toxic in the studied concentrations, confirming its suitability for oral use. The polymers were found to be suitable for the development of compressed tablets loaded with TCM, and their evaluation advocated their appropriateness for the oral delivery of the said drug. 

## Figures and Tables

**Table 1:** Composition of modified-release tablets of TCM.

Ingredients	F1 (PLX) (mg)	F2 (TPLX) (mg)
TCM	4	4
Polymer	45	45
PVP k-30	7.5	7.5
Mg-Stearate	1.5	1.5
Talc	1.5	1.5
Aspartame	3	3
Avicel pH 102	87	87

**Table 2:** Physicochemical properties of PLX and thiolated PLX.

Parameters	PLX ± S.D	TPLX ± S.D
Solubility	Soluble	Soluble
pH (1% Solution)	7.85 ± 0.04	6.68 ± 0.02
Loss on drying (%)	1.4 ± 0.03	11.2 ± 0.04

**Table 3:** Micromeritics analysis of PLX and TPLX.

Parameters	PLX ± S.D (*n* = 5)	TPLX ± S.D (*n* = 5)
Bulk Density (g/cm^3^)	0.466 ± 0.002	0.505 ± 0.003
Tapped Density (g/cm^3^)	0.523 ± 0.002	0.566 ± 0.001
Hausner’s Ratio	1.16 ± 0.017	1.15 ± 0.013
Carr’s Index (%)	10.9 ± 0.0215	10.7 ± 0.020
Angle of Repose (°)	25.78 ± 0.09	21.80 ± 0.201

**Table 4:** Bodyweight, hematology, and blood chemistry of albino rats.

Sr. No	Animals Group Test	Group 1	Group 2	Group 3
**1**	Clinical observations	Nil	Nil	Nil
	Body weight (g)			
	1st day	152	153	175
	3rd day	14	145	174
	7th day	149	148	174
	14th day	150	151	175
**2**	Hematology (Hb (g/dL) (10–15 g/dL)	12.6	13.4	14.2
	Total WBCs (×10^3^ µL)	8.7	7.8	9.6
	RBC’s (×10^6^ µL)	7.11	6.56	6.7
	Platelets (×10^3^ µL)	691	740	934
**3**	Blood chemistry			
	Liver profile			
	AST (U/L)	111	134	148
	ALT (U/L)	35	39	40
	ALP2S (U/L)	128.2	127.1	129
	Bilirubin(mg/dL)	0.05	0.05	0.04
	Total protein (g/dL)	6.2	5.4	6.4
	Renal profile			
	Urea (mg/dL)	25	28	25
	Creatinine (mg/dL)	0.4	0.3	0.3

(All values are expressed as mean; *n* = 3).

**Table 5:** Relative organ weight.

Organ(s)	Control (g)	PLX (g)	TPLX (g)
**Heart**	0.411	0.417	0.413
**Liver**	3.690	3.751	3.735
**Kidney**	0.406	0.399	0.393

**Table 6:** Outcomes of post-compression studies of both PLX- and TPLX-based modified-release compressed tablets of TCM

Post-Compression Studies	F1	F2
Diameter (mm)	1.1 ± 0.086	1.1 ± 0.073
Thickness (mm)	0.5 ± 0.009	0.6 ± 0.147
Weight Variation (%)	Within Limit (±10%)	Within Limit (±10%)
Hardness (N)	4.1–4.25 ± 0.234	4.1–4.26 ± 0.132
Friability (%)	0.34 ± 0.006	0.35 ± 0.005
Disintegration Test (min)	9 ± 0.07	10 ± 0.08
Drug Content (%)	95.89 ± 1.22	97.46 ± 1.85

**Table 7:** Kinetic modeling of release data obtained from dissolution studies of F1 and F2.

Kinetic Models	F1 (PLX)	F2 (TPLX)
Zero Order (R^2^)	0.8935	0.9157
First Order (R^2^)	0.7904	0.760
Higuchi Model (R^2^)	0.8037	0.6878
Korsmeyer Peppas Model (R^2^) (*n*)	0.9723	0.9816
	*n* = 0.61	*n* = 0.65

**Table 8:** Mucoadhesion strength of PLX- (F1) and TPLX- (F2) based compressed tablets.

Formulations	F1	F2
Mucoadhesion Strength (N)	0.99 ± 0.39	2.95 ± 0.35

**Table 9:** The output of the statistical analysis.

Tukey’s Multiple Comparison Test	Mean Diff.	Significant	Summary	*p*-Value
TPLX vs. PLX	11.97	Yes	***	<0.0001
TPLX vs. MP	14.97	Yes	***	<0.0001

*** highly significant values.

## Data Availability

Data is contained within the article or supplementary material.

## Supplementary Materials

The following are available online at https://www.mdpi.com/1999-4923/13/5/693/s1. Supplementary Figure S1: Calibration curve of thiourea, exhibiting good linearity in the selected range of concentration. Supplementary Figure S2: Calibration curve of TCM, showing linearity in the selected concentration range of the drug.

## References

[1] Zarrintaj P., Ramsey J.D., Samadi A., Atoufi Z., Yazdi M.K., Ganjali M.R., Amirabad L.M., Zangene E., Farokhi M., Formela K. (2020). Poloxamer: A versatile tri-block copolymer for biomedical applications. Acta Biomater..

[2] Alawdi S., Solanki A.B. (2021). Mucoadhesive Drug Delivery Systems: A Review of Recent Developments. J. Sci. Res. Med. Biol. Sci..

[3] Leichner C., Jelkmann M., Bernkop-Schnürch A. (2019). Thiolated polymers: Bioinspired polymers utilizing one of the most important bridging structures in nature. Adv. Drug Deliv. Rev..

[4] Bonengel S., Bernkop-Schnürch A. (2014). Thiomers—From bench to market. J. Control. Release.

[5] Prüfert F., Bonengel S., Menzel C., Bernkop-Schnürch A. (2017). Enhancing the efficiency of thiomers: Utilizing a highly mucoadhesive polymer as backbone for thiolation and preactivation. Eur. J. Pharm. Sci..

[6] Hanif M., Zaman M., Qureshi S. (2015). Thiomers: A blessing to evaluating era of pharmaceuticals. Int. J. Polym. Sci..

[7] Davidovich-Pinhas M., Harari O., Bianco-Peled H. (2009). Evaluating the mucoadhesive properties of drug delivery systems based on hydrated thiolated alginate. J. Control. Release.

[8] Iqbal J., Shahnaz G., Dünnhaupt S., Müller C., Hintzen F., Bernkop-Schnürch A. (2012). Preactivated thiomers as mucoadhesive polymers for drug delivery. Biomaterials.

[9] Carvalho F.C., Bruschi M.L., Evangelista R.C., Gremião M.P.D. (2010). Mucoadhesive drug delivery systems. Braz. J. Pharm. Sci..

[10] Prüfert F., Hering U., Zaichik S., Le N.-M.N., Bernkop-Schnürch A. (2020). Synthesis and in vitro characterization of a preactivated thiolated acrylic acid/acrylamide-methylpropane sulfonic acid copolymer as a mucoadhesive sprayable polymer. Int. J. Pharm..

[11] Patel P., Patel H., Panchal S., Mehta T. (2012). Formulation strategies for drug delivery of tacrolimus: An overview. Int. J. Pharm. Investig..

[12] Bhatia M., Ahuja M. (2013). Thiol modification of psyllium husk mucilage and evaluation of its mucoadhesive applications. Sci. World J..

[13] Zaman M., Adnan S., Saeed M.A., Farooq M., Masood Z., Chishti S.A., Qureshi J., Khan R. (2013). Formulation and in-vitro evaluation of sustained release matrix tablets of cellulose based hydrophilic and hydrophobic polymers loaded with loxoprofen sodium. Indo Am. J. Pharma Res..

[14] Bhatia M., Ahuja M., Mehta H. (2015). Thiol derivatization of Xanthan gum and its evaluation as a mucoadhesive polymer. Carbohydr. Polym..

[15] Bravo-Osuna I., Teutonico D., Arpicco S., Vauthier C., Ponchel G. (2007). Characterization of chitosan thiolation and application to thiol quantification onto nanoparticle surface. Int. J. Pharm..

[16] Abdelbary A., Bendas E.R., Ramadan A.A., Mostafa D.A. (2014). Pharmaceutical and pharmacokinetic evaluation of a novel fast dissolving film formulation of flupentixol dihydrochloride. AAPS Pharmscitech.

[17] Mahajan H.S., Tyagi V.K., Patil R.R., Dusunge S.B. (2013). Thiolated xyloglucan: Synthesis, characterization and evaluation as mucoadhesive in situ gelling agent. Carbohydr. Polym..

[18] Yu H., Feng Z.-g., Zhang A.-y., Hou D., Sun L.-g. (2006). Novel triblock copolymers synthesized via radical telomerization of N-isopropylacrylamide in the presence of polypseudorotaxanes made from thiolated PEG and α-CDs. Polymer.

[19] Botham P.A. (2004). Acute systemic toxicity—Prospects for tiered testing strategies. Toxicol. Vitr..

[20] Erum A., Bashir S., Saghir S., Tulain U.R., Saleem U., Nasir M., Kanwal F., Hayat Malik M.N. (2015). Acute toxicity studies of a novel excipient arabinoxylan isolated from Ispaghula (*Plantago ovata*) husk. Drug Chem. Toxicol..

[21] Halim S., Abdullah N., Afzan A., Rashid B.A., Jantan I., Ismail Z. (2011). Acute toxicity study of Carica papaya leaf extract in Sprague Dawley rats. J. Med. Plants Res..

[22] Corti G., Cirri M., Maestrelli F., Mennini N., Mura P. (2008). Sustained-release matrix tablets of metformin hydrochloride in combination with triacetyl-β-cyclodextrin. Eur. J. Pharm. Biopharm..

[23] Pahade M.A.A., Jadhav V.M., Kadam V. (2010). Formulation and development of a bilayer sustained released tablets of isosorbide mononitrate. Stud. Int. J. Pharm. Bio Sci..

[24] Ray D., Prusty A.K. (2010). Designing and in-vitro studies of gastric floating tablets of tramadol hydrochloride. Int. J. Appl. Pharm..

[25] Erum S., Hassan F., Hasan S.M.F., Jabeen S. (2011). Formulation of aspirin tablets using fewer excipients by direct compression. Pak. J. Pharm..

[26] Zhao N., Augsburger L.L. (2005). Functionality comparison of 3 classes of superdisintegrants in promoting aspirin tablet disintegration and dissolution. AAPS PharmSciTech.

[27] El-Kamel A., Sokar M., Naggar V., Al Gamal S. (2002). Chitosan and sodium alginate—Based bioadhesive vaginal tablets. AAPS PharmSci.

[28] Kumari S.D.C., Tharani C., Narayanan N., Kumar C.S. (2013). Formulation and characterization of Methotrexate loaded sodium alginate chitosan Nanoparticles. Indian J. Res. Pharm. Biotechnol..

[29] Saxena A., Tewari G., Saraf S.A. (2011). Formulation and evaluation of mucoadhesive buccal patch of acyclovir utilizing inclusion phenomenon. Braz. J. Pharm. Sci..

[30] Hanif M., Zaman M. (2017). Thiolation of arabinoxylan and its application in the fabrication of controlled release mucoadhesive oral films. Daru J. Pharm. Sci..

[31] Kajdič S., Vrečer F., Kocbek P. (2018). Preparation of poloxamer-based nanofibers for enhanced dissolution of carvedilol. Eur. J. Pharm. Sci..

[32] Han H., Li Y., Peng Z., Long K., Zheng C., Wang W., Webster T.J., Ge L. (2020). A Soluplus/Poloxamer 407-based self-nanoemulsifying drug delivery system for the weakly basic drug carvedilol to improve its bioavailability. Nanomed. Nanotechnol. Biol. Med..

[33] Bernkop-Schnürch A., Scholler S., Biebel R.G. (2000). Development of controlled drug release systems based on thiolated polymers. J. Control. Release.

[34] Bernkop-Schnürch A., Egger C., Imam M.E., Krauland A.H. (2003). Preparation and in vitro characterization of poly (acrylic acid)–cysteine microparticles. J. Control. Release.

[35] Giuliano E., Paolino D., Cristiano M.C., Fresta M., Cosco D. (2020). Rutin-Loaded Poloxamer 407-Based Hydrogels for In Situ Administration: Stability Profiles and Rheological Properties. Nanomedicine.

[36] Zaman M., Hanif M., Sultana K. (2018). Synthesis of thiolated arabinoxylan and its application as sustained release mucoadhesive film former. Biomed. Mater..

[37] Giuliano E., Paolino D., Fresta M., Cosco D. (2019). Drug-loaded biocompatible nanocarriers embedded in poloxamer 407 hydrogels as therapeutic formulations. Medicines.

[38] Zaman M., Bajwa R.I., Saeed S., Hussain M.A., Hanif M. (2020). Synthesis and characterization of thiol modified beta cyclodextrin, its biocompatible analysis and application as a modified release carrier of ticagrelor. Biomed. Mater..

