# Correction: Zaman et al. Synthesis and Evaluation of Thiol-Conjugated Poloxamer and Its Pharmaceutical Applications. *Pharmaceutics* 2021, *13*, 693

**DOI:** 10.3390/pharmaceutics16050690

**Published:** 2024-05-20

**Authors:** Muhammad Zaman, Sadaf Saeed, Rabia Imtiaz Bajwa, Muhammad Shafeeq Ur Rahman, Saeed Ur Rahman, Muhammad Jamshaid, Muhammad F. Rasool, Abdul Majeed, Imran Imran, Faleh Alqahtani, Sultan Alshehri, Abdullah F. AlAsmari, Nemat Ali, Mohammed S. Alasmari

**Affiliations:** 1 Faculty of Pharmacy, University of Central Punjab, Lahore 54000, Pakistan; shafeeq.rahman@ucp.edu.pk (M.S.U.R.); dr.jamshaid@ucp.edu.pk (M.J.); 2 Department of Pharmaceutics, Faculty of Pharmacy, The University of Lahore, Lahore 54000, Pakistan; sadafsaeed14@gmail.com (S.S.); rabiabajwa370@gmail.com (R.I.B.); 3 Oral Biology, Institute of Basic Medical Sciences, Khyber Medical University, Peshawar 59000, Pakistan; saeed.ibms@kmu.edu.pk; 4 Department of Pharmacy Practice, Faculty of Pharmacy, Bahauddin Zakariya University, Multan 60800, Pakistan; fawadrasool@bzu.edu.pk (M.F.R.); abdulmajeed@bzu.edu.pk (A.M.); 5 Department of Pharmacology, Faculty of Pharmacy, Bahauddin Zakariya University, Multan 60800, Pakistan; imran.ch@bzu.edu.pk; 6 Department of Pharmacology and Toxicology, College of Pharmacy, King Saud University, Riyadh 11451, Saudi Arabia; afalasmari@ksu.edu.sa (A.F.A.); nali1@ksu.edu.sa (N.A.); 442106674@student.ksu.edu.sa (M.S.A.); 7 Department of Pharmaceutics, College of Pharmacy, King Saud University, Riyadh 11451, Saudi Arabia; salshehri1@ksu.edu.sa

## Author Name Correction

In the original publication, there was a mistake in one author name, Mohammed Alasmari should be Mohammed S. Alasmari. And accordingly, in the author contributions part, M.A. should be M.S.A.

## Error in Figure

In the original publication, there was a mistake in Figure 6 as published [1]. Unfortunately, the data were not assembled correctly. Now, the below-mentioned Figure 6 is the correct one. 

## Text Correction

In the original publication, there was a mistake in Section 2.3.7 as published [1]. Unfortunately, by mistake, the information (JSM-6490A, Tokyo, Japan) related to the used instruments is not correct. The correct information is EVO LS 10 Zeiss, Germany.

The authors state that the scientific conclusions are unaffected. This correction was approved by the Academic Editor. The original publication has also been updated.

## Figures and Tables

**Figure 6:**
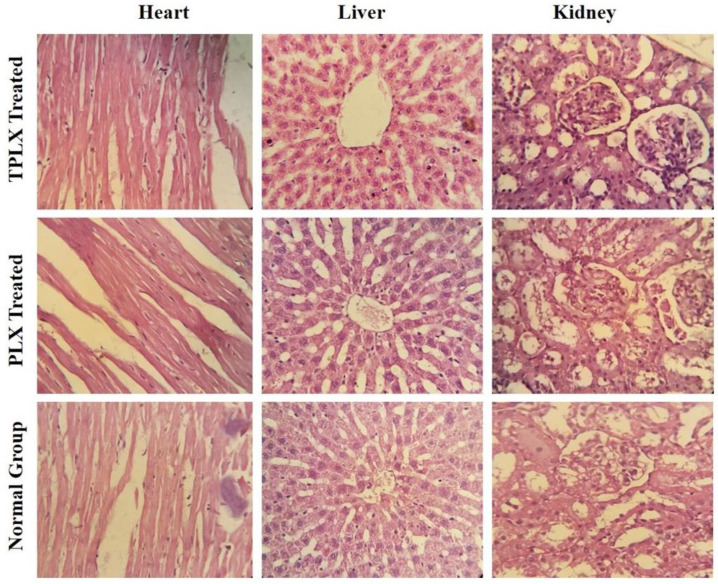
Histopathological evaluation of control group, PLX-treated group, and TPLX-treated group, illustrating the safety profile of modified and unmodified polymers. All the vital organs had normal physiological and anatomical features.
