# Supplementary material for: Synthesis and Evaluation of Thiol-Conjugated Poloxamer and Its Pharmaceutical Applications
Source: Pharmaceutics. 2021 May 11;13(5):693. doi: 10.3390/pharmaceutics13050693 (PMC8151909; doi:10.3390/pharmaceutics13050693)
Supplement: Supplementary file 1 [file pharmaceutics-13-00693-s001.zip › pharmaceutics-1164095-supplementary.pdf]

pharmaceutics

MDPI

Supplementary Materials: Synthesis and Evaluation of Thiol-Conjugated Poloxamer and Its Pharmaceutical Applications

Muhammad Zaman, Sadaf Saeed, Rabia Imtiaz Bajwa, Muhammad Shafeeq Ur Rehman, Saeed Ur Rehman, Muhammad Jamshaid, Muhammad F. Rasool, Abdul Majeed, Imran Imran Faleh Alqahtani, Sultan Alshehri, Abdullah F AlAsmari, Nemat Ali, and Mohammed Alasmari

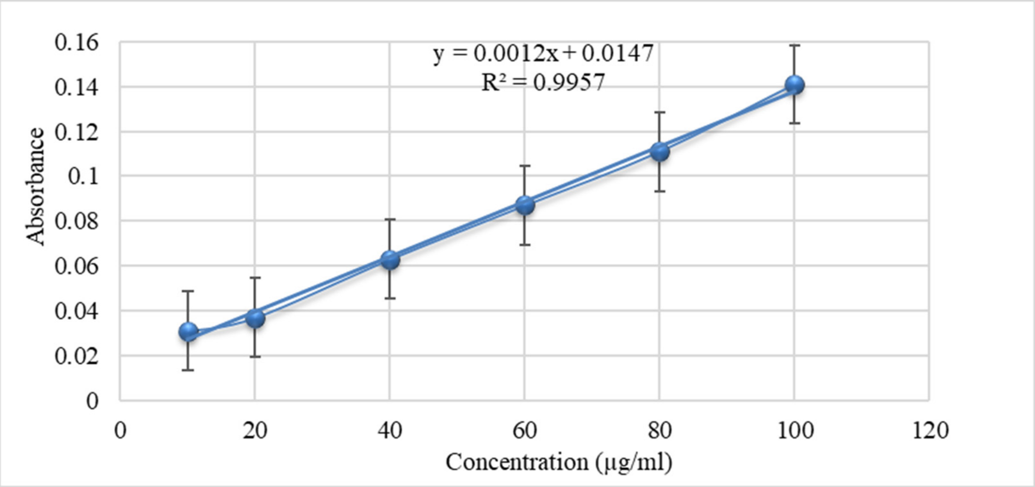

Figure S1. Calibration curve of thiourea, exhibiting a good linearity in the selected range of concentration.

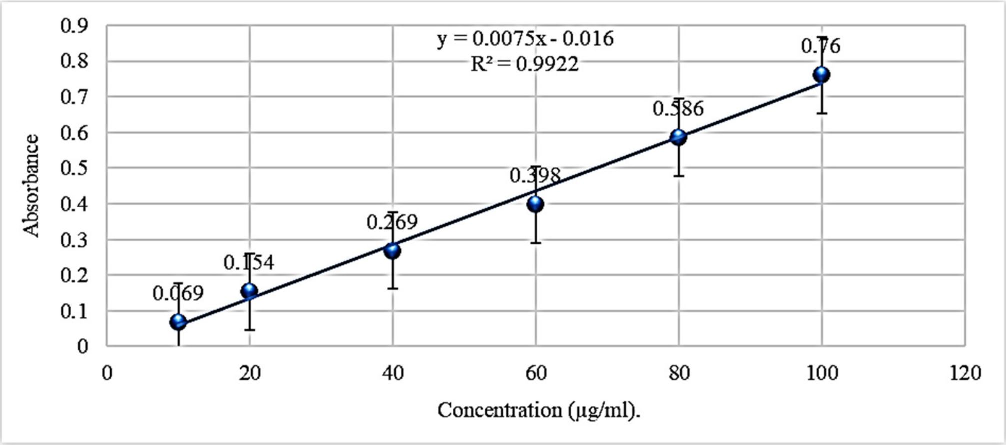

Figure S2. Calibration curve of TCM, showing linearity in the selected concentration range of the drug.
